# Updates on Functional and Morphologic Features via State-of-the-Art Testing in Some Clinically Evident Ocular Diseases

**DOI:** 10.3390/jcm12155052

**Published:** 2023-08-01

**Authors:** Minzhong Yu, Shree K. Kurup

**Affiliations:** Department of Ophthalmology, University Hospitals, Case Western Reserve University, Cleveland, OH 44106, USA

## 1. Introduction

Recent research on functional and morphologic features is relevant to the diagnosis of ocular diseases. The development of new technology in ophthalmology, such as electrophysiology, optical coherence tomography angiography, and optical coherence tomography, are revolutionizing the way details are revealed. It provides a wealth of data for ophthalmologists in the diagnosis of diseases and the evaluation of therapeutic options. This Editorial summarizes the contribution of five original research papers and one review paper from some leading laboratories studying this research topic. They have all provided insight into the important aspects of clinical and basic science research related to the diagnosis and treatment of a wide variety of ocular diseases that include retinal dystrophies, diabetic retinopathy, epiretinal membranes, glaucoma, and amaurosis fugax.

Different electrophysiological modalities can be used to objectively and non-invasively assess the function of different parts of the visual system. A full-field electroretinogram (ffERG) is used to assess the functions of photoreceptors and bipolar cells in rod and cone pathways. Besides the parameters of a-wave and b-wave, oscillatory potentials (OPs) likely reflect the activity initiated by the interactions between bipolar cells, amacrine cells, and ganglion cells, as well as that initiated by a circulatory deficiency in the inner retina. Handheld ERG devices provide a convenient option to test ERG in pediatric patients. With this technique, normal OP values in 132 healthy children aged from 0.3 to 10.6 years old were analyzed [1]. The authors of this study found that the OP implicit times decreased and the amplitudes increased with increasing age, with the implicit times asymptotically approaching the minimums and the amplitudes asymptotically approaching the maximums under 10 years of age, indicating that there was not a significant difference between males and females. According to the extrapolation of the regression curves of the OP1-5 amplitudes **vs.** age, the extrapolated amplitudes of OP1-5 at birth were 1.4, 2.2, 0, and 0.6 µV. These data can be used for clinical pediatric ERG exams as normal values.

The recent development of optical coherence tomography angiography (OCTA), which images the retinal vasculature, has led to more convenience in quantitatively analyzing retinal vessels than when using conventional angiography methods. Using OCTA, the evolution of retinal vessel density (VD) after pan-retinal photocoagulation (PRP) and intravitreal conbercept (IVC) treatment in proliferative diabetic retinopathy (PDR) was analyzed in 55 treatment-naïve PDR eyes (including 29 eyes in the PRP group and 26 eyes in the IVC group) in the study of Zhao et al. [2]. Macular and papillary VD at each follow-up in the PRP group and the IVC group were recorded. According to their data, there was no significant difference in macular VD for superficial capillary plexus (SCP), deep capillary plexus (DCP), choriocapillaris (CC), and papillary VD for radial peripapillary capillary between the two groups at baseline and month 12. It was concluded that, during the 12-month follow-up, both PRP and IVC did not reduce VD in macular, peripapillary, and choroidal capillaries. The authors concluded that both PRP and IVC treatment modalities showed similar protective effects on the macular and papillary capillaries in patients with PDR during the 12-month follow-up.

Optical coherence tomography (OCT) is a well-known technique that allows us to quantitatively map and measure distinctive layers of the retina. In the study of Romano et al. [3], the thicknesses of the central macula, inner layer, retinal nerve fiber layer, ganglion cell layer, inner plexiform layer, inner nuclear layer, outer plexiform layer, and outer nuclear layer were measured by OCT, and the best-corrected visual acuity (BCVA) was recorded in the patients with an idiopathic epiretinal membrane before the operation, as well as 1, 3, and 6 months after the operation. The patients were divided into four groups with different levels of visual recovery, according to the postoperative BCVA improvement. The relationship between the thicknesses of the retinal layers and the four visual recovery patterns were analyzed. It was concluded that different patterns of BCVA recovery were associated with the different thickness changes in different retinal layers [3]. In the review about the relationship between choroidal thickness (CT) measured by OCT and primary open-angle glaucoma (OAG), Verticchio Vercellin et al. investigated the feasibility of using CT as a new biomarker of OAG [4]. While there are reports with conflicting results due to the uncertain relationship between CT and OAG, this review has the potential to inspire future research.

Biallelic variants in the RPE65 gene caused early-onset inherited retinal dystrophy (RPE65-IRD) in a group of patients. In a previous study, Shi et al. described the clinical and genetic findings observed in a Chinese cohort, including 30 patients with RPE65-IRD. They established an association between best-corrected visual acuity (BCVA) and age in 84 Chinese cases (an additional 57 patients, according to the data from the published literature) and discussed fundus features and the natural course of disease in this cohort of patients, concluding that juvenile or young patients present with a comparatively stable BCVA, whereas patients in their third decade of life suffer from a much faster deterioration of vision. These findings are useful in the planning of gene therapy [5].

In a study of the relationship between stroke, myocardial infarction (MI), and amaurosis fugax (AF), the records of 173 patients with AF were retrospectively studied, in which 35.3% of patients were diagnosed with carotid stenosis (10.4% of cases had severe stenosis) [6]. According to the analysis of multivariant regression, there were significant correlations between carotid stenosis and ischemic heart disease, as well as between age and gender. In addition, 9.2% of patients had a stroke after a diagnosis of AF, in which 68.8% of whom suffered from carotid artery stenosis; 1.7% of patients were diagnosed with MI after AF; and all of them did not present with carotid artery stenosis. Furthermore, 2.3% of these patients were reported as presenting with central retinal artery occlusion after AF, and all of them had carotid artery stenosis. With these data, the authors concluded that, after an AF attack, a high incidence of internal carotid artery stenosis occurred, which was significantly associated with stroke. Finally, there was no increase in the incidence of MI and CRAO after AF, in which only CRAO was associated with carotid artery stenosis.

Overall, these six articles on this research topic provide updates on the functional and morphologic features that can be obtained with state-of-the-art testing in some ocular fundus diseases, and they have practical and research applications.
